# Solitary Extrapleural Fibrous Tumor in Salivary Glands: Our Experience—Case Series and Literature Review

**DOI:** 10.3390/diagnostics12112688

**Published:** 2022-11-04

**Authors:** Ciro Emiliano Boschetti, Rita Vitagliano, Gianmaria Imola, Nicola Cornacchini, Maria Luisa Colella, Gianpaolo Tartaro, Giuseppe Colella

**Affiliations:** Oral and Maxillofacial Surgery Unit, Multidisciplinary Department of Medical-Surgical and Dental Special- 8 ties, University of Campania “Luigi Vanvitelli”, Via Luigi de Crecchio, 6, 80138 Naples, Italy

**Keywords:** solitary extrapleural fibrous tumor, parotid gland, submandibular gland, sublingual gland, salivary gland, malignancies, solitary extrapleural fibrous tumor of the head and the neck, benign tumors

## Abstract

(1) Background: Extrapleural solitary fibrous tumors (ESFTs) are rare oncological entities occurring in the head and neck, and even more so in the salivary glands. The clinical presentation and histologic features are usually unspecific, resulting in frequent misclassification. As an unusual tumor, ESTFs have an unpredictable clinical behavior. (2) Methods: We present two clinical cases referred to our Maxillofacial Surgery Unit for the onset of a symptomless mass involving, in one case, the parotid gland, and in the other case, the sublingual gland. (3) Results: Solitary fibrous tumors could be considered as neoplasms with intermediate biological behavior that are not entirely predictable on the basis of morphological features, as these are mostly still unknown. However, a few histologic, immunohistochemical, and imaging features, such as a hypodense signal at the T1 sequence in an MRI, or positivity for CD34, bcl2, and CD99, and the NAB2-STATS6 fusion gene, could be useful for an early differential diagnosis of ESTFs. (4) Conclusions: All patients were alive at follow-up with no evidence of disease. Surgical management should always be considered as the first choice for oncological radicality, and clinical behavior should always be defined with the help of the study of radiological and anatomopathological features.

## 1. Introduction

In 1931, Kempler and Rabin described a series of localized solitary pleural tumors arising from subpleural areolar tissue, and this was thought to be the first description of what we now refer to as the solitary fibrous tumor (SFT) [1]. Since 1931, this entity has been referred to as fibrous mesothelioma, subpleural fibroma, and a localized fibrous tumor of pleura. They divided primary pleural tumors into two categories: diffuse mesothelioma and localized mesothelioma.

They proposed that diffuse mesothelioma was mesothelial in origin, while mesothelioma (now known as solitary fibrous tumor) was of submesothelial origin [2].

The histogenesis of SFTs is controversial; little evidence in the literature supports a mesothelial origin, but modern immunohistochemistry and ultrastructural features demonstrate that SFTs are not mesothelial but involve undifferentiated mesenchymal cell histogenesis.

The SFT has mesenchymal cell origins and arises from primitive fibroblast-like cells in connective tissue. As a matter of fact, spindle cells tumors bear a close resemblance to this localized pleural tumor, and they have also been described in a variety of anatomic sites that are not lined by the mesothelium [3].

SFTs have been reported in numerous extrathoracic anatomical regions, such as the orbits, oral cavity and salivary glands, thyroid, larynx, trachea, intracranial and spinal cord meninges, liver, pancreas, adrenal gland, kidney, female genital tract, prostate, pelvis, breast, soft tissue of extremities, and periosteum of bone.

Currently, it is estimated that about 30–40% of reported SFTs are extrapleural. Among the extrathoracic sites, the orbit and the soft tissue of the extremities (muscles and subcutaneous tissues) are the most commonly reported anatomical regions [4].

As a rare and unusual oncologic entity, ESFTs’ immunohistochemical, radiological, and laboratory features are unspecific, and they may have a clinical presentation as an indolent symptomless mass, and for this reason, ESFTs could be miscategorized and not immediately diagnosed. There are some common features that are used to reach a correct definitive diagnosis, such as the immunoreactivity of antibodies directed against CD34, as well as, in few cases, CD99 and Bcl-2; under a radiologic point of view, the best imaging technique to typify the ESFT is the MRI, as usually this tumor shows a hypodense signal at the T1 sequence, and T2-weighted sequences are useful for the visualization of the fibrous component of the lesions, which has a characteristically hypodense signal in T2.

T1-weighted and CT images are also important for the evaluation of the hemosiderin component and calcifications, which are difficult to evaluate in T2 sequences. The enhancement of SFTs on postcontrast CT scans and MR imaging is commonly heterogeneous, a typical appearance also called “geographic paper”. Usually, laboratory features are completely normal, but occasionally hypoglycemia is reported, which is probably related to IGF’s secretion by ESFTs’ neoplastic cells.

Surgical resection represents the first treatment choice; when this is not practical, radiation therapy could be considered after evaluating the tumor’s behavior and anatomopathological diagnosis or the margins of excision of the ESFT.

Recurrencies are uncommon, but a close follow-up is always recommended.

In our experience, a 52-year-old man and a 42-year-old woman were referred to the Maxillofacial Surgery Unit for the onset of a symptomless mass involving, in the first case, the parotid gland, and in the second case, the sublingual gland [5].

## 2. Detailed Case Description

**Figure 1:**
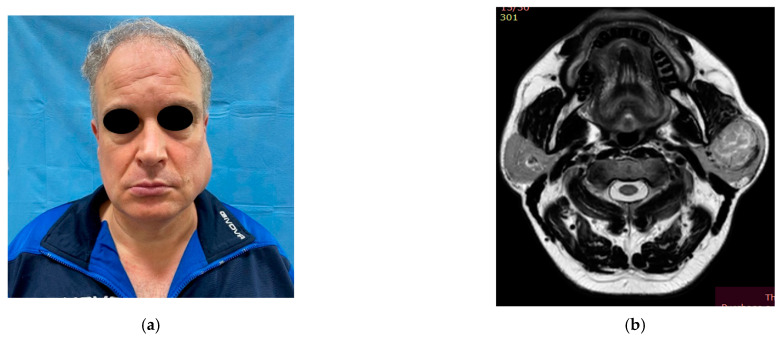
(**a**) Our 52-year-old patient affected by a symptomless mass of the left parotid gland (**b**) The T.C. scan of the facial massif performed on the patient.

A 52 year-old man presented in October 2021 to the U.O.C. of the Maxillofacial Surgery Unit at the “Università degli studi della Campania- L. Vanvitelli with a non-ulcerated, non-exophytic, hyperemic, large, firm mass without everted or rolled margins that was apparently painless, with a maximum diameter of 7 × 2.3 cm that extended from the left preauricular region to the middle-parotid region (Figure 1a).

The patient reported the development and swelling had taken place for about a year during the pandemic. Following his primary care physician’s recommendation, an echo-guided FNAC of the lesion was performed for the patient, which described a cytologic result of a blood background and a monomorphic population of basal cells, which was poorly differentiated and predominantly organized in three-dimensional solid groups. No metachromatic matrix or cribriform aspects were observed, and the primary diagnosis was compatible with a basaloid cell neoplasm or a spindle cell myoepithelioma and classified following the Milan System for Reporting Salivary Glands Cytopathology (MSRSGC) as a category IV-B or SUMP (salivary gland neoplasm of uncertain malignant potential).

A soft tissue echography was carried out for the patient in September 2021; it was performed with a multifrequency linear transducer. On the left, at the level of the palpable and visible tumefaction, appearing as well-circumscribed and apparently capsulated in the parotid region, there was a coarse structural alteration with a homogeneously hypoechogenic pattern with multiple shoots and septa of medium echogenicity affecting the entire gland. This alteration showed multiple vascular signals on color Doppler inspection that were classified as a mixed ultrasound pattern. Lymph nodes with a reactive appearance and a maximum diameter of 1 cm were described at upper and lower parotid peripolar sites. There was no evidence of dilatation of Stenone’s duct.

In September 2021, a CT scan was carried out for the facial massif, performed in basal conditions with a multidetector spiral technique. This examination was performed without contrast, upon request of the patient, and there was evidence of asymmetry of the parotid loggias due to a marked volumetric increase in the left one, which was moreover altered by artifacts from dental implants on the maxillary upper; reactive lymph nodes were represented as peri- and subcentimetric in the left submental, submandibular, and laterocervical stations. (Figure 1b).

In October 2021, the echo-guided FNAC of the left parotid region was repeated.

The morphological result shows direct smears against a blood background, a modest cellularity consisting of a cohesive cell population in small groups. The cell population was small and almost devoid of cytoplasm, with basaloid differentiation. The diagnostic category for cytology in salivary glands, according to the Milan System, was confirmed to be IV-B SUMP.

The patient was operated on and dismissed from our U.O.C. in November 2021.

The surgical technique involved a classical Blair incision with subsuperficial musculo-aponeurotic system dissection in the left parotid region. The mucocutaneous flap was raised to the anterior and inferior borders of the gland. The posterior branch of the greater auricular nerve was carefully identified and preserved. Soft tissue dieresis extended to the posterior belly of the digastric, sternocleidomastoid was found, and the facial nerve trunk and its branches were found to be isolated and preserved (Figure 3a). Left superficial parotidectomy was performed, with facial nerve branches found to be intertwining and adhered to the neoformation. The deep portion of the tumor was removed, which was strongly vascularized and attached to the masseter muscle. A superficial musculoaponeurotic system (SMAS) platysma flap was sutured to the parotid capsule, and parenchyma was used to replace soft tissue volume loss and reduce the incidence of Frey syndrome [6]. Hemostasis was controlled, a suction drain was placed at the parotidectomy site, and the parotid lodge was closed primarily with resorbable sutures.

Two hours after surgery, the patient had an early postoperative complication caused by abnormal bleeding. He was reoperated on for a wound revision and evacuation of the hematoma to control the bleeding sites. During the operative procedure, the previous incision was used as the surgical access. The bleeding surgical sites were washed with saline solution so the vessel in the active bleeding phase could be found. Clots were aspirated, and a procedure to control the hemostasis was performed; a new suction drain was positioned, and the parotid lodge was closed primarily with resorbable sutures.

**Figure 2:**
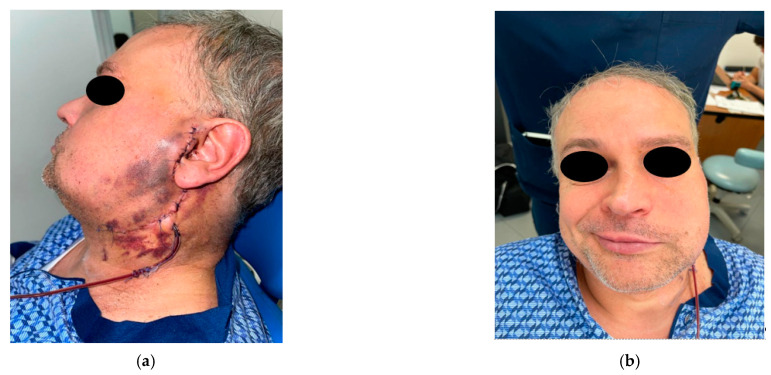
(**a**) Immediate postoperative complication: hematoma. (**b**) Immediate postoperative complication: facial nerve deficit.

At the end of the surgery, the patient was transferred to an intensive care unit for 24-h close systemic monitoring, in accordance with our protocol for surgical patients after an early complication (Figure 2a).

The postoperative therapy was based on intravenous antibiotic therapy (Clavulanic acid plus Amoxicillin, 2 g in 1 vial intravenously 2 times daily), pain relief therapy (Paracetamol, 1000 mg in one vial intravenously 3 times daily), corticosteroid therapy (Betamethasone, 4 mg in one vial intravenously 2 times daily), and proton-pump inhibitors (Omeprazole, 40 mg in one vial intravenously 1 time daily).

The patient was discharged home 3 days after surgery with the antibiotic, pain relief, and corticosteroid therapies. We also indicated Kabat therapy [7,8] (Figure 2b), and the patient was advised to not expose the surgical wound to the sun and to apply 50+ SPF sunscreen.

**Figure 3:**
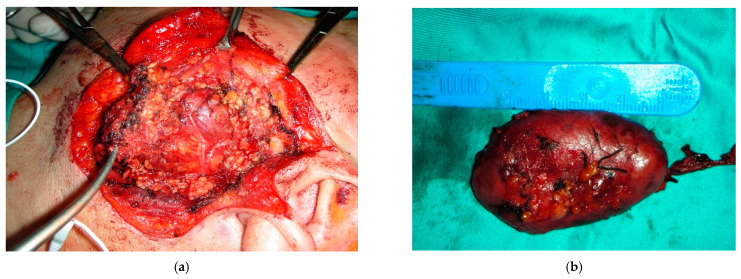
(**a**) Intraoperative photos of the surgical excision. (**b**) The anatomopathological specimen.

The postoperative histopathologic study had a macroscopic description of a nodular specimen of 4 × 2.5 × 2 cm with an adherent portion of salivary gland with dimensions of 4 × 3 × 2 cm, showing a yellowish lobulated injection that appeared apparently capsular, and, once serrated, had a compact whitish appearance. The microscopic description showed a well-circumscribed mesenchymal proliferation that was predominantly hypercellular with sparse hyalinized areas. Focal areas with a pseudocystic pattern were present. The neoplasm predominantly consisted of a epithelioid cell population, with round oval nuclei and cytoplasm with indistinct borders; in some areas, the cellular component took on a more spindle-like appearance with pleomorphism. In the context of proliferation, there was a conspicuous proportion of small-to-medium-caliber vessels, with a wide lumen; in the medium-caliber vascular component, the walls were hyalinized. Histiocytes phagocytosing hemosiderin pigment were present in the hyalinized areas. Erythrocyte extravasation was observed. Necrosis was absent. Poor mitotic activity (up to 2 mitoses/10 HPF) was also noted. Immunohistochemical studies showed positivity for STAT6, bcl, and CD99 and negativity for smooth muscle actin, calponin, CD34, CD31, ERG, p63, GFAP, S100, CKAE1AE3, and CK7. The serous-type salivary gland parenchyma present in the specimen appeared unscathed (Figure 3b). A small 3 mm periglandular lymph node was found at the site of sinus histiocytosis.

The histological diagnosis reports morphologic and immunophenotypic aspects consistent with an extrapleural solitary fibrous tumor.

**Figure 4:**
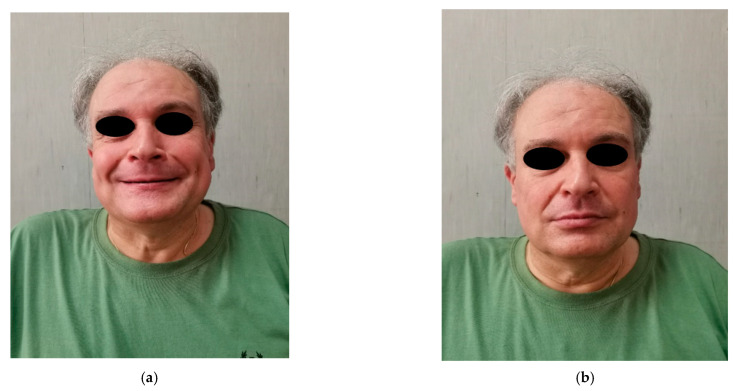
(**a**,**b**) Postoperative photos of our patient at 6-month follow-up, after Kabat therapy, with recovery of the facial nerve deficit.

At the 6-month follow-up, after Kabat therapy, the patient recovered a facial nerve deficit of about 85% and did not show signs of recurrency (Figure 4a,b).

**Figure 5:**
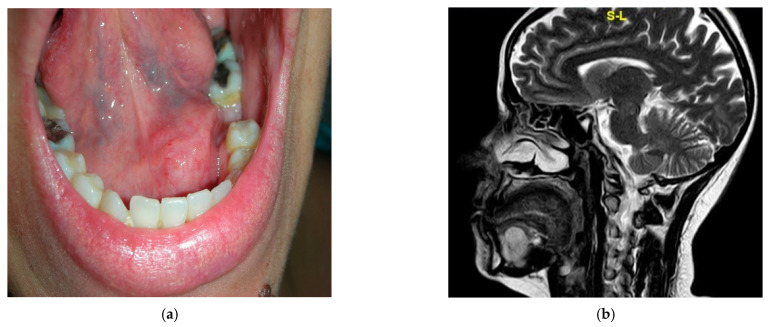
(**a**) Multiplanar MRI performed on the patient. (**b**) Preoperative images of our patient’s mass.

A 42-year-old woman was referred to the Maxillofacial Surgery Unit in February 2018 for a non-ulcerated, non-exophytic, normochromic submucosal mass without everted or rolled margins involving the left oral pelvis, mainly the left sublingual area. Intraoral examination revealed a firm nodule about 3 cm in diameter (Figure 5a). A multiplanar MR scan was performed and confirmed a well-demarcated and capsulated mass adhered to the sublingual gland.

The lesion was shown to be homogeneous and isointense on T1-weighted images and heterogeneous and mildly hyperintense on T2 images; there was no evidence of bone infiltration, and cervical nodes with swelled dimensions were found (Figure 5b).

An FNAC of the lesion was performed in March 2018 for the patient, showing scanty cellularity represented by spindle cells with plump nuclei with an inconclusive diagnosis of mesenchymal proliferation.

Therefore, a surgical approach to the mass was suggested to a multidisciplinary committee and then planned.

**Figure 6:**
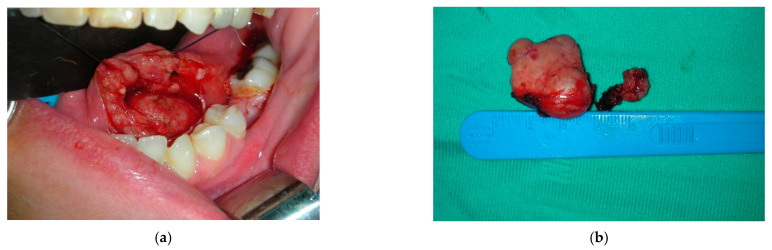
(**a**) Intraoperative photos of the surgical excision. (**b**) The anatomopathological specimen.

The surgical technique involved an intraoral excision to the left floor of the mouth, from the incisive region to the molar region, along the major axis of the neoformation. Exposition of the lesion was performed, and after an intraoperative examination, the mass appeared to be adhered to the surrounding tissues, especially to the sublingual gland that was completely removed. The lingual nerve was not infiltrated by the tumor, and for this reason was spared. At the end of the surgery, the patient stayed in our unit for 48-h close systemic monitoring, in accordance with our protocol for surgical patients at risk of an early complication (Figure 6a).

The postoperative therapy was based on intravenous antibiotic therapy (Clavulanic acid plus Amoxicillin, 2 g in one vial intravenously 2 times daily), pain relief therapy (Paracetamol, 1000 mg in one vial intravenously 3 times daily), corticosteroid therapy (Betamethasone 4 mg in one vial intravenously 2 times daily), and proton-pump inhibitors (Omeprazole 40 mg in one vial intravenously 1 time daily).

The patient was discharged home 3 days after surgery with the antibiotic, pain relief, and corticosteroid therapies.

Gross examination showed a nodule with a diameter of 2.8 cm and a whitish, solid, and vaguely multilobulated cut and capsulated appearance; however, microscopically, the specimen was constituted by a major component of neoplastic proliferation arranged in a multinodular pattern with alternation in the hypocellular and hypercellular areas, and showed oval-to-spindle-shaped cells with undefined cellular borders and bland nuclear features, embedded in a fibrillar and myxoid stroma, and a dense cellular population with very scant stroma. In these fields, the cells showed an oval shape, scant cytoplasm, undefined cellular borders, a more irregular chromatinic pattern, and relevant mitotic activity (about five mitoses on 10 high-power fields). The neoplasm also showed well-marked and capsulated borders. Immunohistochemistry was performed and included the following antibodies: CK (clone AE1/AE3), CD34 (clone QBEnd/10), CD99 (clone O13), bcl2 (clone 124), S100 (clone SP127), desmin (clone DE-R-11), ALDH1 (clone 44/ALDH1), and STAT6 (clone EP325) (Figure 6b).

The mass was positive for CD34, CD99, and ALDH1 and focally positive for bcl2. Interestingly, CD99 stained the hypercellular areas more intensely. Stains with STAT6, S100, and desmin were negative. A diagnosis of a malignant extrapleural solitary fibrous tumor was confirmed.

The postoperative combined multiparametric MR-PET confirmed the total removal of the tumor and showed no other location of the neoplasm.

A follow-up of 2 years was performed, and no recurrence was evidenced.

A literature search was also performed in order to compare our findings with other reports. The research was carried out on the PubMed database, for which we identified articles up to July 2022. Article language was limited to English using database-supplied filters. No limitations on study designs were included. The keywords used and combined with Boolean operators were as follows: “solitary extrapleural fibrous tumor”, “parotid gland”, “submandibular gland”, “sublingual gland”, “salivary gland”, “malignancies”, “solitary extrapleural fibrous tumor of the head and the neck”, and “benign tumors”. The literature search identified 10 articles. Six articles were excluded due to having no relevance to this case (ESFTs in pediatric age, ESFTs of the ethmoid, ESTFs of the orbital region, ESTFs of the cheek, ESTFs of the auditory canal, recurrent extrapleural solitary fibrous tumors). Four articles were selected for full-text evaluation. The selection process identified three articles that were compatible with our criteria, which will be discussed in the Section 3.

## 3. Discussion

The etiology of extrapleural SFTs is currently not defined, and no association has been demonstrated with smoking habits or asbestos exposure. Numerous karyotypic aberrations, including both numerical and structural abnormalities, have been reported in extrapleural ESFTs.

Several studies reported a gain of chromosomes 5, 8, 13, and 21; others reported a complete loss or partial deletion of chromosomes 1, 6, 9, 13, 15, 17, 18, and X [9].

Another important aspect is the demonstration of the NAB2-STATS6 fusion gene, which encodes a chimeric protein; correspondingly, the C-terminal repressor domain of NAB2 is replaced by a highly variable portion of the STAT6 protein.

There is not a specific age of appearance, but they are more frequent in the fifth decade, with no gender difference.

The majority of ESFTs have an indolent course, and they have a low risk of recurrence after their surgical treatment. The 10-year overall survival in this case is about 90%.

A total of 10–25% of ESFTs may have local recurrence or will present as a disseminate disease. These patients and those who are not eligible for the surgical treatment or cannot resort to surgery have a poor prognosis [10].

In 2013, WHO classified SFTs as fibroblastic–myofibroblast neoplasms with intermediate, rarely metastasizing, biological behavior. The clinical symptoms depend on the anatomical site of development, but they usually occur as slowly growing masses, being often an asymptomatic incidental finding or related to the pressure generated on the adjacent anatomical structures [11].

It is possible, if rare, to have hypoglycemia, probably due to the secretion of IGF by the neoplastic cells [12].

The most useful diagnostic technique used to evaluate solitary fibrous tumors, as mesenchymal-cell-derived tumors involving mainly soft tissues, is magnetic resonance imaging (MRI); most of the tumors (about 80%) show a hypodense signal at the T1 sequence.

T2-weighted images are extremely helpful in identifying the fibrous component of the lesions, which has a characteristically hypodense signal in T2.

T1-weighted and CT images are useful to evaluate two of SFTs’ distinguishing elements: a hemosiderin component and calcifications, which are really difficult to evaluate in T2 sequences, due to their low signal.

In solitary fibrous tumors with a low fibrous content, T2-weighted scans have an intermediate-to-hyperintense signal. Lesions with a diffuse myxoid loose stroma component also appear hyperintense in T2-weighted scans. The contrast enhancement is heterogeneous (with a characteristic “geographic paper” appearance) in most cases [13].

Due to the infrequency of ESFTs, little is known about their pathological characteristics and clinical features. For these reasons, ESFT salivary glands tumors are a diagnostic challenge for every oral and maxillofacial and head and neck surgeon [14].

The management of ESFTs, as a rare oncological entity, should always be discussed among a multidisciplinary committee for an early diagnosis, as the radiological, the anatomopathological, and oncological features could be easily unrecognized or misclassified.

The first clinical case that we presented had histologic, immunohistochemical, and imaging features comparable to the ones reported in the literature; we report a characteristic feature of that case, the intense vascularization of the neoformation, which led to postoperative complications and reintervention. However, in the second clinical case, histologic, immunohistochemical, and imaging features were seen, which are completely new in the literature, and comparable to other sublingual ESFT cases.

The reported solitary fibrous tumors had a benign course. About 10–15% have aggressive or malignant behavior; cases of fibrous tumors mutated into high-grade sarcomas are reported in the literature [15].

Solitary fibrous tumors could usually be considered as neoplasms with intermediate biological behavior that is not entirely predictable on the basis of morphological features; however, according to the modern anatomopathological literature, a few morphological features are associated with a higher rate of local recurrence and more frequent distant metastases, such as infiltrative margins, pleomorphism, hypercellularity, a mitotic index greater than 4/10, high-power fields, and necrosis [16]. If an extrapleural solitary fibrous tumor responds to a minimum of three of these morphological features, it is defined as a “malignant ESFT”. A definite diagnosis and the determination of predictable behavior require histological postoperative examination of the neoplasm; as a matter of fact, in our cases, in the preoperative examination, echo-guided FNAC was needed for a solitary fibrous tumor, which was classified according to the Milan System for Reporting Salivary Glands Cytopathology (MSRSGC) as a category IV-B or SUMP salivary gland neoplasm of uncertain malignant potential, without any further characterization.

In one of our cases, we had an ESFT with hypercellularity, nuclear atypias, and a mitotic index of five mitoses per 10 high-power fields, so it could be defined as a “malignant ESFT”. Meanwhile, in the other case of ESFT, we found poor mitotic activity with a mitotic index of two mitoses per 10 high-power fields, pleomorphism, hypercellularity without nuclear atypias, and well-circumscribed margins, without necrosis; thus, we could define it as a “benign ESFT”. ESFTs arising from the parotid gland parenchyma are rare, with only 40 cases (including the current one) reported in the literature since 1995. Most reported parotid gland SFTs (PG-SFTs) show benign behavior; meanwhile, malignant ESFTs are even rarer in salivary glands, and the current literature shows only three case reports, to the best of our knowledge [6].

The first is an article written in 1997 by J. Sato et al. with the title “Solitary fibrous tumor of the parotid gland extending to the parapharyngeal space”, and described a case of an ESFT of the deep lobe of the parotid gland that invaded the parapharyngeal space, with a so-called histological “patternless pattern”, with a successful resection by an external approach. The patient was recurrence-free for 1 year after the surgical operation [17].

The second article, which is compatible with our domain, was written in the 2001 by Alawi et al. with the title “Solitary Fibrous Tumor of the Oral Soft Tissues A Clinicopathologic and Immunohistochemical Study of 16 Cases”, and describes 16 cases of ESTF oral soft tissue, including two salivary-gland-related cases with immunohistochemical, clinical, and radiological features similar to our first described case of “benign ESTF” [18].

The third article is compatible with our outcome domain and was written in 2002 by Muñoz Guerra et al., with the title “Solitary fibrous tumor of the parotid gland: A case report”, and describes a case of a 37-year-old woman with a symptomless mass in the right parotid gland without any evidence of facial nerve paralysis with same radiological, clinical, and histopathological features of our first case. In this article, the ESTF is described as a completely rare entity, with 12 cases in the literature described for the major salivary glands, and only 6 cases for the parotid glands. Additionally, in this case, there was a “patternless” histological pattern, and a very interesting element of the article was the differential diagnosis of soft tissues and the immunohistochemistry profile of CD34-positive tumors of soft tissue, such as solitary fibrous tumors, hemangiopericytoma, spindle cell lipoma, Kaposi’s sarcoma, peripheral neural sheath tumors, or malignant fibrous histiocytoma. This article also highlights that the complete behavior of these neoplasms in the parotid gland is still unknown, and notes that as we have few anatomopathological indicators that can be used to establish the most adequate surgical plan, there are still no defined reliable guidelines for tumor prognosis [19].

## 4. Conclusions

SFTs of the salivary glands are extremely rare oncologic entities that most often occur in middle-aged patients, with no predominance in a certain gender, who may have been living with a symptomless mass lesion for a long time. Diagnosis of SFTs is based on classical histologic and immunophenotypic features with CD34, bcl-2, and CD99 immunoreactivity, which allows for distinction and separation from other tumors in the differential diagnosis, but we hope that in the future, clinical and radiological features could also lead to a better early diagnosis. The presence of hypercellularity, relative pleomorphism, and a mitotic index of 2/10 HPF suggest a possible increased aggressiveness of the neoplasm.

Given the rarity and unpredictability of the biological behavior of these tumors, it is necessary to schedule long-term follow-ups, as there is a possibility that patients will develop recurrences or distant metastases even many years after surgery [3]. Follow-up in our experience suggests a good prognosis whether the tumors are histologically benign or malignant when managed by complete surgical excision, without recurrence in the short term after radical surgical management.

## Data Availability

Data are available upon reasonable request from the corresponding author (R.V.).
